# Implementation of Mini-FLOTAC in Routine Diagnosis of Coccidia and Helminth Infections in Domestic and Exotic Birds

**DOI:** 10.3390/vetsci8080160

**Published:** 2021-08-09

**Authors:** João Lozano, Cristina Almeida, Ana Cláudia Victório, Pedro Melo, João Paulo Rodrigues, Laura Rinaldi, Giuseppe Cringoli, Lídia Gomes, Manuela Oliveira, Adolfo Paz-Silva, Luís Madeira de Carvalho

**Affiliations:** 1 CIISA—Centro de Investigação Interdisciplinar em Sanidade Animal, Faculdade de Medicina Veterinária, Universidade de Lisboa, Avenida da Universidade Técnica, 1300-477 Lisbon, Portugal; anaklaudiarv@gmail.com (A.C.V.); lidia@fmv.ulisboa.pt (L.G.); moliveira@fmv.ulisboa.pt (M.O.); madeiradecarvalho@fmv.ulisboa.pt (L.M.d.C.); 2 ExoClinic—Clínica Veterinária de Aves e Exóticos, Quinta de Santo António, 1495-049 Miraflores, Portugal; cristinaalmeida@mail.com; 3 Vetnatura—Serviços Veterinários, Lda., Calçada de Palma de Baixo, 1600-176 Lisbon, Portugal; vetnatura@gmail.com; 4 Quinta dos Plátanos, Rua do Serrado, 2205-306 Pego, Portugal; jpbuenorodrigues@hotmail.com; 5 Veterinary Parasitology and Parasitic Diseases, Department of Pathology and Animal Health, Faculty of Veterinary Medicine, University of Naples Federico II, Via Federico Delpino, 80137 Naples, Italy; laura.rinaldi@unina.it (L.R.); giuseppe.cringoli@unina.it (G.C.); 6 Control of Parasites Research Group (COPAR, GI-2120), Department of Animal Pathology, Faculty of Veterinary, University of Santiago de Compostela, 27142 Lugo, Spain; adolfo.paz@usc.es

**Keywords:** poultry, exotic birds, gastrointestinal parasites, Mini-FLOTAC, Portugal

## Abstract

Mini-FLOTAC (MF) has recently been proposed for the fecal quantification of gastrointestinal (GI) parasites in birds due to its higher sensitivity and precision in comparison with the McMaster method. The current research aimed to test the use of MF in routine diagnosis of coccidia and helminth infections in several domestic and exotic bird collections in Portugal. Between July 2020 and April 2021, a total of 142 fecal samples from organic layers, peacocks and ratites were collected in four Portuguese bird collections and processed using MF and fecal cultures to identify and calculate GI parasite shedding and prevalence. The McMaster method was also used to compare the shedding levels obtained for both quantitative techniques. MF’s relative sensitivity and specificity were also assessed, using McMaster as the reference technique. The implementation of MF resulted in an average *Eimeria* spp. shedding higher in peacocks from bird collection 2 (502 OPG), followed by peacocks from collection 1 (107 OPG) and organic layers (24 OPG) and peacocks from collection 3 (9 OPG). Peacocks were also positive for *Capillaria* spp., *Trichostrongylus tenuis* and *Strongyloides* *pavonis*, whereas ostriches and emus were infected by *L. douglassii*. The MF protocol for exotic animals and the McMaster method did not differ significantly for each parasitic agent and bird species, and MF achieved relative sensitivities and specificities higher than 70% for Galliform *Eimeria* spp., peacock helminths and ratites’ *L. douglassii* infections. Higher *L. douglassii* EPG values were identified using the MF protocol for exotic species (2 g of feces/38 mL of sucrose solution), followed by McMaster 2/28, MF 5/45 and MF 2/18. The use of MF allowed for obtaining different intestinal parasitic populations in several bird species and locations, and MF 2/38 is globally proposed as the most suitable protocol for bird fecal samples as an alternative to the McMaster method in the diagnosis of avian intestinal parasitic infections.

## 1. Introduction

Free-range poultry and captive wild birds are commonly housed in limited areas with high animal stocking and access to the environment and co-habit with other bird species and wild avifauna, consequently being exposed to a wide variety of generalist or host-specific gastrointestinal (GI) parasites, namely coccidia and helminths, which are still responsible for severe health and economic concerns in poultry farms and zoological collections worldwide [1,2,3,4,5,6].

Coccidia infections in Galliformes from free-range farms, zoos and public gardens can reach prevalence and shedding values of up to 80% and 15,000 oocysts per gram of feces (OPG), respectively [1,6,7,8,9,10]. Avian helminth infections are also a reality in traditional free-range farms, private exotic collections and public gardens, with ascarids (e.g., *Ascaridia galli*), heterakids (e.g., *Heterakis gallinarum* and *H. isolonche*), *Trichostrongylus tenuis*, *Strongyloides* spp. and *Capillaria* spp. being the most prevalent and pathogenic nematodes in domestic and exotic Galliformes such as poultry and peacocks [1,7,8,10,11].

Birds of the order Struthioniformes, such as ostriches, emus and rheas, are also frequently housed in zoos and occasionally in farms across the globe, and GI parasitism by helminths is of the most clinical importance in these birds. Infections caused by *Libyostrongylus douglassii* are noteworthy, which is the most common and pathogenic nematode in ostriches and other ratites, being responsible for rotten stomach disease. There are also other species of *Libyostrongylus*, such as *L. magnus* and *L. dentatus*, the latter having only been recorded in North America thus far. *Codiostomum struthionis* is also commonly found in ratites, inhabiting the distal cecum and upper rectum of adult birds and occasionally being responsible for hemorrhagic processes and oedema in the cecum’s mucosa [2,12,13,14,15].

Over the past seven years, Mini-FLOTAC (MF) has been used in routine parasitological diagnosis in several animal species, and most of the studies concluded that this technique is a good alternative to the traditional McMaster technique, allowing simultaneous identification of helminth eggs and coccidia oocysts with relatively higher sensitivity, accuracy and precision. Established in the Unit of Parasitology and Parasitic Diseases of the Department of Veterinary Medicine and Animal Production (University of Naples Federico II, Naples, Italy), the manufacturer proposes three MF protocols for animals, namely for small animals (e.g., dogs and cats), herbivores (e.g., ruminants and horses) and exotic species (e.g., birds and reptiles), which involve different fecal dilutions and detection limits [16,17,18].

Despite the constant annual increment in studies involving the use of MF in several animal species, its implementation in epidemiological studies in birds has been extremely scarce thus far, and there is a lack of consensus regarding the optimal protocol for the diagnosis of coccidia and helminth infections in these hosts. However, recent studies with MF in birds have demonstrated its potential in the diagnosis of common avian coccidia and nematodes, with some achieving sensitivities of up to 100% [19,20,21,22].

The current research aimed to implement and optimize MF in the routine diagnosis of GI parasitic infections in several domestic and exotic bird species from different collections across Portugal and compare the resulting shedding levels with the traditional McMaster method.

## 2. Materials and Methods

### 2.1. Bird Collections and Fecal Samplings

**Figure 1:**
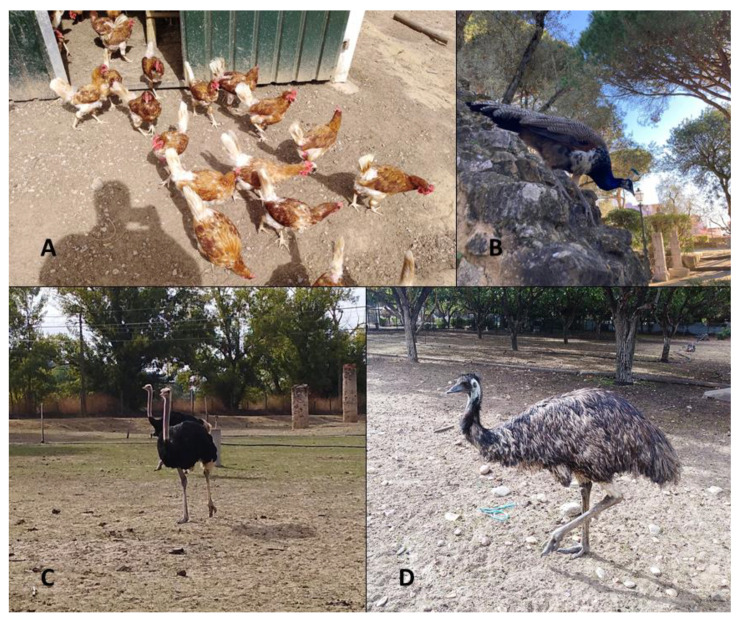
Domestic and exotic birds selected for this study: (**A**) organic layers, (**B**) peacocks, (**C**) ostriches and (**D**) emus (originals).

Between July 2020 and April 2021, a total of 142 fecal samples from organic layers (*Gallus gallus domesticus*) (*n* = 46), peacocks (*Pavo cristatus*) (*n* = 68), ostriches (*Struthio camelus*) (*n* = 9) and emus (*Dromaius novaehollandiae*) (*n* = 19) (Figure 1) were collected from a poultry farm and several exotic bird collections in Portugal. The sampling sites were located in the Lisbon and Santarem districts, comprising bird collection 1 (Lisbon, 38°42′50.241″ N 9°8′2.182″ W), bird collection 2 (Lisbon, 38°45′30.44″ N 9°9′23.83″ W), bird collection 3 (Abrantes, 39°26′52.595″ N 8°10′24.949″ W) and a poultry farm (Lourinhã, 39°13′54.373″ N 9°17′2.235″ W), whose species and housing conditions are summarized in Table 1.

Fresh fecal samples were randomly collected after excretion to the environment, deposited in individual plastic bags and then immediately transported in a cooling bag to the Laboratory of Parasitology and Parasitic Diseases of the Faculty of Veterinary Medicine, University of Lisbon, where they were stored in a refrigerator (4–5 °C) for 1 week, processed and analyzed.

This research followed the daily activity of the selected farm and bird collections in strict collaboration with the owners and assistant veterinarians, and no interferences were made in the regular health management of all collections.

### 2.2. Coprological Techniques

All samples were processed and analyzed with the Mini-FLOTAC technique, aiming to calculate gastrointestinal parasites’ shedding (eggs and oocysts per gram of feces—EPG and OPG, respectively), whereas fecal cultures were used for taxonomical identification of coccidia and helminths with environmental larval development. The McMaster method was also used in each sample to compare average EPG and OPG data with MF [16,22,23].

#### 2.2.1. Mini-FLOTAC

**Figure 2:**
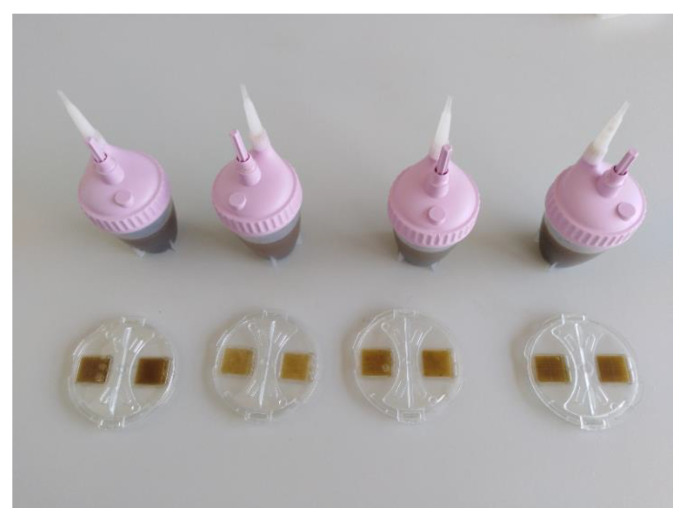
Mini-FLOTAC and Fill-FLOTAC devices used for quantitative coprology (original).

The Mini-FLOTAC protocol followed the guidelines proposed by the manufacturer for exotic species (MF 2/38): 2 g of feces was added to the corresponding Fill-FLOTAC device and mixed with 38 mL of saturated sucrose solution (specific gravity 1.2); then, the fecal suspension was transferred to the (previously assembled) reading chamber and left for 10 min to rest on the lab bench before rotating the top piece of the reading chamber (Figure 2). Coccidia oocysts and helminth eggs were identified and counted in a light microscope (100×), using detection limits of 10 OPG and 10 EPG, respectively [16,22].

The three Mini-FLOTAC protocols proposed by the manufacturer for small animals (2 g/18 mL of saturated sucrose solution, specific gravity 1.2), exotic species (2 g/38 mL) and herbivores (5 g/45 mL) were also tested in 8 ostrich fecal samples positive for the nematode *L. douglassii*, with shedding values higher than 1000 EPG, using detection limits of 5, 10 and 5 EPG, respectively, aiming to compare the resulting EPG levels between the MF and McMaster protocols [16].

The relative sensitivity of Mini-FLOTAC was calculated as the percentage of true positive reads (TP) in the sum of false negative (FN) and TP reads, while Mini-FLOTAC’s relative specificity was calculated as the percentage of true negative reads (TN) in the sum of false positive (FP) and TN reads [21,22]. These parameters were calculated for Galliform coccidia, peacock helminths and ratites’ *L. douglassii*, assuming the McMaster method as the reference technique due to its historic and frequent use for quantitative copromicroscopy in most parasitology laboratories [19,24,25].

#### 2.2.2. McMaster

For the McMaster method, 2 g of each fecal sample was mixed with 28 mL of saturated sucrose solution (specific gravity 1.2), and the filtered suspension was transferred to a McMaster slide. Parasitic forms were identified and counted under a light microscope (100×), using a detection limit of 50 OPG and 50 EPG [23].

#### 2.2.3. Fecal Cultures

Fecal cultures for oocyst sporulation were performed only with samples positive for coccidia, using 5–10 g of each fecal sample, which were placed on Petri dishes with potassium dichromate (2%) and incubated for 1 week at 26 °C. Sporulated oocysts were identified based on their size and the number of sporocysts inside to the genus level. Fecal cultures for helminths were only conducted with samples positive for nematodes with environmental larval development using 5–10 g of each fecal sample, which were placed inside plastic cups and incubated for 2 weeks at 26 °C. Infective larvae (L3) obtained from ostrich samples were analyzed in terms of their morphology and measures and compared with current reports in the literature for different species of *Libyostrongylus* and *Codiostomum* [23,26,27,28].

### 2.3. Statistical Analysis

The software Microsoft^®^ Excel^®^, for Microsoft 365 MSO (Microsoft Corporation, Redmond, WA, USA, 2021), was used for data storage and table and chart editing, and the software GraphPad InStat^®^, version 3.0 for Windows (GraphPad Software, San Diego, CA, USA, 2021), was used for statistical analysis.

Data normality was assessed with the Kolmogorov–Smirnov test, and for every group of animals and quantitative technique, EPG and OPG data failed the normality test (*p* < 0.0001). These results determined the use of the following non-parametric tests: Kruskal–Wallis test for *Eimeria* OPG comparison between each sampling site; Mann–Whitney test for helminth EPG comparison between peacocks from collections 1 and 2 and between ostriches and emus from collection 3; Wilcoxon matched pairs test for Mini-FLOTAC and McMaster comparison in Galliform *Eimeria* spp., peacock helminths and ratites’ *L. douglassii*. For the Mini-FLOTAC optimization trial in ostrich samples, EPG data passed the Kolmogorov–Smirnov test (*p* > 0.10), and the results obtained for each protocol were compared using the Tukey–Kramer multiple comparisons test. A significance level of *p* < 0.05 was used for all statistical tests.

## 3. Results

### 3.1. Epidemiological Results with Mini-FLOTAC in Domestic and Exotic Birds

The current research with Mini-FLOTAC revealed an overall *Eimeria* spp. prevalence higher in peacocks from bird collection 3 (43%), followed by organic layers (41%) and peacocks from bird collections 1 and 2 (29% and 25%, respectively). The overall coccidia prevalence in Galliformes was 35%. However, the average *Eimeria* spp. shedding was higher in peacocks from bird collection 2 (502 OPG) in comparison with peacocks from collection 1 (107 OPG) and organic layers (24 OPG) and peacocks from collection 3 (9 OPG), which were statistically significant differences (*p* < 0.0001). Additionally, the average *Eimeria* OPG in all Galliformes reached 160 OPG (Table 2).

Helminth species were identified in all bird collections except for the poultry farm, in which *Eimeria* spp. oocysts were the only intestinal parasitic forms found. Collections 1 and 2 exhibited different helminthic populations: birds from collection 1 were positive for *Capillaria* spp. (14%) and *S. pavonis* (14%), and birds from collection 2 were positive for *T. tenuis* (8%) and *S. pavonis* (4%). Helminth EPG was higher in peacocks from collection 1 (145 EPG) in contrast to collection 2 (66 EPG), despite the fact that their differences were not significant (*p* = 0.58), and peacocks from collection 3 were not found infected by any helminth.

**Figure 3:**
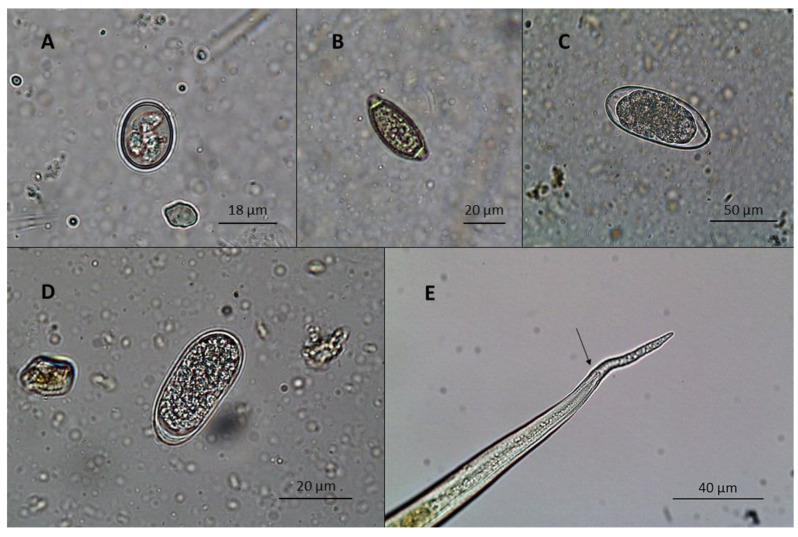
(**A**) *Eimeria* sp. oocyst; (**B**) *Capillaria* sp. egg; (**C**) *T. tenuis* egg; (**D**) *S. pavonis* egg; (**E**) tail and sheath end of *L. douglassii* L3 identified in emus, highlighting the typical tail-end knob format (black arrow) (originals).

Ratites from collection 3 were infected by *L. douglassii*, with frequencies of 100% and 32% and average shedding levels of 2731 and 60 EPG for ostriches and emus, respectively, with the EPG results differing significantly between ratite species (*p* < 0.0001) (Figure 3).

### 3.2. McMaster and Mini-FLOTAC Comparison

The McMaster and Mini-FLOTAC techniques resulted in similar average shedding values for *Eimeria* spp. in Galliformes (188 and 160 OPG, respectively) and *L. douglassii* in ratites (1647 and 1396 EPG, respectively), and their differences were not significant (*p* = 0.17 and *p* = 0.67 for Galliformes and ratites, respectively). Helminths’ average shedding values in the aggregate community of peacocks from collections 1 and 2 reached 16 and 105 EPG for McMaster and MF, respectively, and the techniques also did not differ significantly (*p* = 0.08) (Table 3).

The relative sensitivity of the MF technique for Galliform coccidia, peacock helminths and *L. douglassii* in ratites reached 86%, 86% and 100%, respectively, and the relative specificity of MF was 70%, 100% and 87%, respectively.

**Figure 4:**
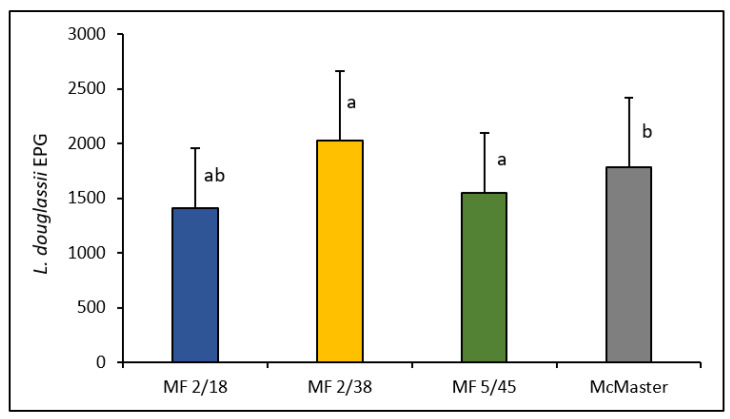
*L. douglassii* average EPG values and standard deviations in Mini-FLOTAC for small animals (MF 2/18), exotic animals (MF 2/38) and herbivores (MF 5/45) and McMaster. a: significant differences between MF protocols; b: significant difference between MF 2/18 and McMaster.

The MF optimization trial in ostrich fecal samples positive for *L. douglassii* resulted in higher EPG levels for the MF 2/38 protocol (2028 ± 636 EPG), followed by McMaster (1788 ± 635 EPG), MF 5/45 (1553 ± 543 EPG) and MF 2/18 (1410 ± 550 EPG) (Figure 4). Differences were statistically significant between the pairs MF 2/18–MF 2/38 (*p* < 0.001), MF 2/18–McMaster (*p* < 0.05) and MF 5/45–MF 2/38 (*p* < 0.01). For the pairs MF 5/45–MF 2/18, MF 2/38–McMaster and MF 5/45–McMaster, no significant differences were observed (*p* > 0.05 for each pair).

## 4. Discussion

The current research focused on the innovative implementation of Mini-FLOTAC in the routine diagnosis of gastrointestinal parasitic infections in several species of domestic and exotic birds in different housing conditions and kept for different purposes. The use of this technique for epidemiological purposes revealed different parasitic scenarios in each sampling site regarding the intestinal parasites identified and their respective shedding levels and frequencies.

Coccidia belonging to the genus *Eimeria* were the most prevalent intestinal parasites in organic layers and peacocks from all locations, which confirms this group of parasites as ubiquitous in several species of domestic and exotic Galliformes [6,8,23,28].

Organic layers from the poultry farm had a moderate *Eimeria* spp. prevalence (41%), similar to previous research in free-range/organic broiler and layer flocks, which highlights the importance of regular monitoring of birds’ health status, namely through periodic blood and fecal samplings for parasitological analysis [6,9,10]. These birds were not subjected to any antiparasitic drug program since organic animal production is extremely regulated in the European Union, and it is forbidden to use antiparasitic drugs for prophylactic purposes, which therefore poses a higher risk of developing parasitic diseases in organic flocks. However, the overall *Eimeria* spp. shedding in these birds was considerably low (24 OPG), which can be explained by the advanced age of the flock (16 months) and the low stocking density, reflecting a potential equilibrium between the parasite and the host immune system, which is common in older domestic birds [6,9,29,30].

The period of the year in which sampling took place may have also influenced the resulting *Eimeria* spp. shedding in organic layers, as the Mediterranean summer season is frequently long, dry and hot, commonly ending in late October, which offers adverse environmental conditions for oocysts’ survival and sporulation in the soil, thus limiting their dissemination between birds, as concluded by other authors in different countries and climatic conditions [9,10,29,30]. This research did not identify helminths in samples from organic layers, unlike other publications regarding parasitological assays in birds kept in organic, free-range or backyard conditions [6,31,32]. Possible explanations may be the low stocking density and advanced age of the flock, which limit the dissemination of eggs among birds—particularly relevant in parasites with direct life cycles (e.g., ascarids) [23,33]. The adverse impact of direct sunlight and high temperatures frequently recorded during summertime in the Mediterranean region may have also affected the survival of helminth eggs in the environment [31].

Regarding the three peacock communities included in this research, Lisbon bird collections 1 and 2 had birds infected by coccidia and helminths, while samples from collection 3 were only positive for *Eimeria* spp. Bird collection 2 showed the highest recorded coccidia shedding level in peacocks (502 OPG) in comparison with bird collections 1 and 3. The high coccidia shedding level in peacocks from collection 2 might have been influenced by the season in which sampling took place, since springtime is the breeding period for peacocks, leading to an increase in the number of chicks which are more prone to be infected by coccidia than older birds, as well as being a season in the Mediterranean region normally characterized by moderate temperatures and relative humidity and sporadic episodes of intense rainfall, which are optimal conditions for oocyst sporulation in the soil and horizontal transmission among birds [7,9,10,29,30].

Differences were also observed in the helminth populations of peacocks from collections 1 and 2. *Capillaria* eggs were only identified in collection 1, *T.* *tenuis* infections were only detected in peacocks from collection 2 and *S.* *pavonis* eggs were identified in samples from both collections. These results allow for confirming the susceptibility of peacocks to helminths [7,8], both by ingestion of infective forms directly from feces or soil, or through their intermediate hosts, and by being exposed to wild free-ranging avifauna, which often leads to episodes of cross-transmission [5]. All intestinal parasites identified in these communities are the first of their kind to be reported in ornamental peacocks from Portuguese public gardens.

Ostriches and emus from bird collection 3 were infected by *L. douglassii*, which is the most pathogenic helminth in ratites, and this is in accordance with other findings in ostriches in South America [2,26,34], Asia [35], Oceania [14,36,37,38], Africa [39] and Europe [12,13].

Despite commonly being considered specific to ostriches, *L. douglassii* was first identified 21 years ago in emus kept in Sweden [12], which, at the time, suggested the potential cross-transmission of this helminth between different ratite species. Since then, no other research has identified this nematode across the ratites group, but it has been suggested that infections would likely be infrequent in these bird species [14]. The current study detected infections by *L. douglassii* in emus, reaching a prevalence of 32%, and confirmed the cross-transmission of the host–species barrier. However, the shedding levels were very low (60 EPG) when compared to ostriches (2731 EPG), and their differences were statistically significant, which allows us to conclude that ostriches are indeed more prone to be infected by this helminth than emus are. It must be noted that in bird collection 3, these two ratite species were normally separated in distinct parks, and infections were only recorded in emus when they were mixed with ostriches in the same area, which explains the low average shedding level of *L. douglassii* in emus and the host-specificity of this nematode, while revealing that cross-transmission for this helminth might indeed occur in the ratites group [12]. These results in emus also have implications in zoo animal management, since it is advisable to not mix different species of ratites in the same areas to avoid serious outbreaks of Libyostrongylosis in these birds.

The overall shedding and prevalence of *L. douglassii* in this study were higher than noted in previous research regarding this helminth in ratites, which is an interesting result since the majority of samples were collected during the winter season; low temperatures could therefore potentially limit helminth infections in these birds. The results from this study suggest the survival and maintenance of the infectious capacity of *L. douglassii* L3 larvae on soil during wintertime, as revealed by previous research conducted in Scandinavia [40].

The implementation of the Mini-FLOTAC exotic animal protocol in bird fecal samples allowed the identification of the most common species of gastrointestinal parasites in the selected domestic and exotic birds, and both the relative sensitivities and specificities of this technique for all groups of intestinal parasites reached values higher than 70%. The use of MF in the detection of *Eimeria* spp. infections in Galliformes reached a relative sensitivity of 86%, similarly to previous research regarding the comparison of the MF and McMaster techniques in the detection of free-range poultry *Eimeria* spp. infections [22].

Samples from ratites, which had an average shedding higher than 1000 EPG, reached a relative sensitivity of 100%, meaning that for this level of shedding, there was no difference between both techniques and each positive sample averaged a true positive read with MF. These results are in accordance with previous research using the MF and McMaster methods for the detection of ascarid eggs in chicken feces, whose authors concluded that MF tended to be more sensitive than McMaster only at lower EPG levels, while the difference between them was not significant for shedding levels higher than 50 EPG [21].

Comparison of McMaster and MF regarding Galliform *Eimeria* spp., peacock helminths and ratites’ *L. douglassii* shedding allowed for concluding that these techniques reached similar OPG and EPG results, which did not differ significantly regardless of the parasitic agent. These results are similar to previous research with MF and McMaster in the detection of avian *Eimeria* spp., in which the authors also did not identify significant differences between these two techniques, using the same protocol [16,22].

The MF optimization trial using ostrich samples positive for *L. douglassii* allowed us to observe that the protocol established by the manufacturer for exotic animals resulted in higher shedding values in comparison with the other MF protocols (small animals and herbivores) and the McMaster method. The mean EPG values obtained with the MF exotic animal protocol differed significantly from the protocols for small animals and herbivores, but it did not differ from the McMaster method. One of the reasons for obtaining higher and statistically significant EPG results with the exotic animal protocol in comparison with the other MF protocols may have been due to the conjugating effect of a clearer reading and a higher multiplication factor as a result of the dilution used (1:20) [16,19]. On the other hand, since the dilutions were quite similar, the lack of significance between the McMaster technique and the MF 2/38 protocol may be explained by the difference in the multiplication factor between these techniques. Even though the McMaster technique revealed a poorer resolution and ranked second in terms of mean EPG, its multiplication factor of 50 EPG was enough to counter an eventual significant difference with MF 2/38.

The fact that both MF 2/38 and MF 5/45 did not differ significantly when compared to the McMaster method reflects the identical results these three techniques can achieve. Furthermore, since MF 2/38 achieved the highest EPG levels when compared to the other MF protocols and the McMaster protocol and differed significantly from the MF 5/45 protocol, it allows us to conclude that MF 2/38 can indeed be globally considered the best alternative to the McMaster method.

## 5. Conclusions

The current research accurately implemented Mini-FLOTAC in the routine diagnosis of gastrointestinal parasites in several domestic and exotic bird species and allowed us to identify different parasitic scenarios in the selected bird communities in Portugal, being the first European report in terms of using this technique in different avian species kept in captivity for different purposes, namely for egg production or ornamental exhibition.

The types of bird species, age amplitude of flocks, access to the environment, exposure to wild avifauna and season were potential key factors responsible for the wide diversity of intestinal parasitic species identified in this research. Galliformes were mainly infected by coccidia belonging to the genus *Eimeria* and helminths such as *Capillaria* spp., *T.* *tenuis* and *S.* *pavonis*, with differences between organic layers and peacocks, and this is the first national report of gastrointestinal parasitism in peacocks from public gardens. Moreover, this study identified *L. douglassii* infections in ratites, which is the most pathogenic helminth in this group of birds, and the cross-infection and breaking of the host–species barrier for this helminth was confirmed in emus, being the first report in more than 20 years.

Comparison of the MF and McMaster techniques in Galliformes, peacocks and ratites allowed us to conclude that the MF exotic animal protocol is the best alternative to the McMaster method in birds, and therefore, the current study proposes this MF protocol for routine diagnosis of avian gastrointestinal parasitosis.

## Figures and Tables

**Table 1:** Sampling periods, bird species, quantity of samples and outdoor areas.

Period	Collection	Species	No. of Birds	Age	Samples	Outdoor Area
October–December 2020	Bird collection 1 (Lisbon)	Peacocks	20	3 months–9 years	29	9600 m^2^
April 2021	Bird collection 2 (Lisbon)	Peacocks	40	3 months–19 years	25	6000 m^2^
September 2020–February 2021	Bird collection 3 (Abrantes)	Ostriches	2	4 years	9	50,000 m^2^
		Emus	6	7–14 years	19	
		Peacocks	3	3–6 years	14	6000 m^2^
July–November 2020	Poultry farm (Lourinhã)	Organic layers	200	16 months	46	1700 m^2^

**Table 2:** Epidemiological results obtained with Mini-FLOTAC for each bird species.

Collection	Bird Species	GI Parasites	OPG|EPG (Min–Max)	Prevalence (%)
Bird collection 1	Peacocks	*Eimeria* spp.	107 (0–750)	29
		Helminths	145 (0–2000)	14 (*Capillaria* spp.)
				14 (*S. pavonis*)
Bird collection 2	Peacocks	*Eimeria* spp.	502 (0–1800)	25
		Helminths	66 (0–1000)	8 (*T. tenuis*)
				4 (*S. pavonis*)
Bird collection 3	Ostriches	*L. douglassii*	2731 (500–5700)	100
	Emus		60 (0–420)	32
	Peacocks	*Eimeria* spp.	9 (0–30)	43
Poultry farm	Organic layers	*Eimeria* spp.	24 (0–300)	41
Average *Eimeria* spp., Galliformes			160 (0–1800)	35
Average Helminths, Peacocks			70 (0–2000)	15
Average Helminths, Ratites			1396 (0–5700)	66

**Table 3:** Mini-FLOTAC and McMaster shedding data obtained for each bird group.

Bird Groups	GI Parasites	Mini-FLOTAC EPG|OPG (Min–Max)	McMaster EPG | OPG (Min–Max)
Galliformes	*Eimeria* spp.	160 (0–1800)	188 (0–5100)
Peacocks (Collections 1 and 2)	Helminths	105 (0–2000)	16 (0–400)
Ratites	*L. douglassii*	1396 (0–5700)	1647 (0–10,000)

## Data Availability

Not applicable.
